# Ring finger protein 12 activates AKT signalling to promote the progression of liver cancer by interacting with EGFR

**DOI:** 10.1111/jcmm.17757

**Published:** 2023-05-02

**Authors:** Chengpeng Yu, Dean Rao, Tiantian Wang, Jiaqi Sheng, Enjun Lv, Long Zhang, Xun Lu, Jingjing Yu, Huifang Liang, Jia Song, Wenjie Huang

**Affiliations:** ^1^ Hepatic Surgery Center Tongji Hospital, Tongji Medical College, Huazhong University of Science and Technology Wuhan China; ^2^ Hubei Key Laboratory of Hepato‐Pancreato‐Biliary Diseases Tongji Hospital, Tongji Medical College, Huazhong University of Science and Technology Wuhan China; ^3^ Department of Hepato‐Pancreato‐Biliary Surgery Ganzhou People's Hospital of Jiangxi Province (Nanchang University Affiliated Ganzhou Hospital) Ganzhou Jiangxi China

**Keywords:** EGFR internalization, liver cancer, PI3K‐AKT signalling, RNF12

## Abstract

Liver cancer is one of the most common solid tumours, and ranks as the third leading cause of cancer‐associated mortality around the world. This study has linked RNF12 to the pathogenesis of liver cancer. Based on the analysis of patient samples and database data, high RNF12 expression was found in liver cancer, in correlation with worse clinicopathological features and a poor prognosis. Meantime, RNF12 could promote the progression of liver cancer in vitro and in vivo*.* Mechanistically, RNF12 could interact with EGFR and decrease the internalization of EGFR to activate EGF/EGFR signalling. In addition, PI3K‐AKT signalling takes part in the regulation of liver cancer cell proliferation and migration of RNF12. And AKT inhibitor MK2206 could reverse RNF12‐mediated cellular proliferation and migration in liver cancer. The possibility of the physical interaction between RNF12 and EGFR might lay a foundation to develop intervention strategies for liver cancer prevention and therapy.

## 1 INTRODUCTION

Liver cancer is one of the most common solid tumours, and ranks as the third leading cause of cancer‐associated mortality around the world.^1^ Liver cancer patients are often diagnosed at an advanced stage, which results in a poor prognosis. It is therefore crucial to obtain a better understanding of the development of liver cancer to enable early diagnosis and better treatment. Multiple factors promote the occurrence and development of liver cancer, such as alcohol abuse, aflatoxin B1 exposure, hepatitis B virus, hepatitis C virus and metabolic syndrome.^2^, ^3^, ^4^, ^5^ Under the influence of these factors, many genes and signalling pathways are dysregulated in liver cancer, such as Wnt/β‐catenin, AKT/mTOR, P53/cell cycle regulation and EGF/EGFR signalling.^6^, ^7^, ^8^ Understanding these dysregulated signalling pathways is crucial for the early diagnosis and treatment of liver cancer.

Ring finger protein 12 (RNF12), also known as RLIM, is an X chromosome‐linked E3 ubiquitin ligase that targets LIM homeodomain, which results in the degradation of a cofactor of LIM protein.^9^ Meantime, RNF12 also functions as a co‐transcription factor to bind to DNA to regulate the expression of downstream genes.^10^ Physiologically, RNF12 has been reported to be involved in the inactivation of X chromosome, embryonic development, cell differentiation and maintenance of neural function.^11^ Recently, increasing research showed that RNF12 was involved in the development of cancer.^12^ In glioblastoma, RNF12 was reported to be highly expressed and related to poor prognosis of patients.^13^ Meantime, RNF12 interacted with and ubiquitinated RB1 to induce its degradation, which activated the MAPK signalling pathway to promote the proliferation of glioblastoma.^13^ In breast cancer, the high expression of RNF12 was reported to be related to the distant metastasis of breast cancer patients.^12^ In addition, RNF12 interacted with and ubiquitinated SMAD7 to cause its degradation and promote TGF‐β‐induced breast cancer metastasis.^12^ However, RNF12 interacted with c‐myc and decreased its transcriptional activity to inhibit the cellular proliferation of osteosarcoma cells.^14^ These studies revealed that RNF12 played complex roles in regulating different signalling pathways that contributed to cancer progression. However, the roles of RNF12 in liver cancer have not been explored in detail. In this study, we found that RNF12 activated the PI3K‐AKT signalling pathway by interacting with EGFR to promote liver cancer cell proliferation and invasion in vitro and in vivo. In agreement with these findings, high RNF12 expression was found in patient samples of liver cancer and correlated with a poor prognosis.

## 2 PATIENTS AND METHODS

### 2.1 Patients

Human liver tumour tissues and corresponding adjacent normal tissues were obtained from 116 liver cancer patients who performed hepatic resection from 16 February 2012 to 1 April 2014 at the Hepatic Surgery Center, Tongji Hospital of Huazhong University of Science and Technology (HUST) (Wuhan, China). Ethical approval was obtained from the Ethical Committee of Tongji Hospital, Huazhong University of Science and Technology (HUST). All patients provided written informed consent for use of their tissue specimens. The methodologies of this study conformed to standards set by the Declaration of Helsinki. The diagnosis of liver cancer was confirmed by pathologists and their clinical stages were determined based on the BCLC classification. The patients of liver cancer with the following conditions were excluded: (1) patients ≤18 or ≥70 years of age; (2) patients with a history of preoperative anticancer radiotherapy or chemotherapy, biological, immune and traditional Chinese medicine; (3) patients with incomplete postoperative follow‐up data; (4) patients with a history of another organ malignancy or systemic immune disease. The time from the first operation to death or recurrence/metastasis was defined as overall survival (OS) and disease‐free survival (DFS), respectively.

### 2.2 Western blot analysis and immunohistochemistry

Western blot analysis and immunohistochemistry was conducted as described previously.^15^ The immunohistochemical score was based on the degree of staining (0–3 points) and the positive rate (0–4 points), and then multiplied to obtain a comprehensive score (0–12 points). The staining intensity was scored by the staining depth of the cells under the microscope: 0 point for non‐staining, 1 point for light yellow, 2 points for brown yellow and 3 points for brown; The positive rate was scored by the cell positive ratio: 0%–5% for 0 point, 6%–25% for 1 point, 26%–50% for 2 points, 51%–75% for 3 points and >75% for 4 points. The following antibodies were used: anti‐RNF12 (#H00051132, Abnova), anti‐GAPDH (KC‐5G4, Kang‐Chen BioTech Co., Ltd.), anti‐EGFR (#C74B9, Cell Signaling Technology), anti‐phospho‐EGFR (Y1173) (#53A5, Cell Signaling Technology), anti‐phospho‐EGFR (Y1068) (#D7A5, Cell Signaling Technology), anti‐p38 (#8690, Cell Signaling Technology), anti‐phospho‐p38 (1:1000, #9215, Cell Signaling Technology), anti‐AKT (#4691, Cell Signaling Technology), anti‐phospho‐AKT (Ser473) (#4060, Cell Signaling Technology), anti‐JNK (#9258, Cell Signaling Technology), anti‐phospho‐JNK (Thr183/Tyr185) (#700031, Invitrogen), IgG (sc‐2027, Santa Cruz Biotechnology), anti‐Flag‐tag (#F1804, Sigma‐Aldrich), anti‐HA‐tag (#H6908, Sigma‐Aldrich), IgG (sc‐2025, Santa Cruz Biotechnology), and MK2206 (S1078, Selleck).

### 2.3 Cell lines and culture conditions

Huh7, HepG2 and HEK 293 cells were obtained from the China Center for Type Culture Collection (CCTCC). The HLF, HLE, Hep3B and MHCC97H cells were maintained in our laboratory. The culture condition was in the environment of 37°C, 5% CO_2_ and saturated humidity.

### 2.4 Plasmids and lentiviral vectors

The pcDNA3.1, Plvx‐Luc‐IRES‐NEO, pcDNA3.1‐AKT and pcDNA3.1‐EGFR plasmids came from our laboratory. The pEnter‐RNF12 plasmid was purchased from WZ Biosciences lnc. The pLKO.1‐TRC cloning vector (Plasmid # 10878), plenti‐CMV‐Puro, pMD2.G (Plasmid #12259) and psPAX2 (Plasmid #12260) vectors were purchased from Addgene. To obtain RNF12 overexpressing cell lines, RNF12 cDNA was cloned into plenti‐CMV‐Puro vector. Then pLenti‐RNF12, psPAX2 and pMD2.G were co‐transfected into HEK293 cells to obtain lentiviral. Lentiviral was used to affect target cells, which were then selected by puromycin (5 μg/mL) to obtain RNF12‐overexpressing cell lines. To obtain RNF12 knockdown cell lines, three target sequences and one non‐targeting sequence (negative control, NC) were cloned into the pLKO.1 vector. The sequences of shRNA oligo pairs for RNF12 and AKT are listed in Table S1. PLKO.1‐shRNF12, psPAX2 and pMD2.G were co‐transfected into HEK293 cells to obtain lentiviral. Lentiviral was used to affect target cells, which were then selected by puromycin (5 μg/mL) to obtain RNF12 knockdown cell lines.

### 2.5 Immunoblotting and co‐immunoprecipitation (co‐IP)

Immunoblotting and co‐IP were conducted as described previously.^16^ Briefly, IP lysis buffer containing proteinase inhibitor was used to lyse the cells on ice. The lysates were incubated with protein G agarose for 2 h on a rotating platform, and the lysates were immunoprecipitated with specified antibodies at 4°C overnight. On the second day, the lysates were incubated with protein G agarose for 1 h and then washed once with IP lysis buffer and washing buffer (pH 7.4, 1.0% NP‐40, 300 mM NaCl, 25 mM Tris–HCl, 1.0 mM EDTA) five times. Then, 2× SDS‐PAGE loading buffer was used to elute the beads for immunoblotting analysis.

### 2.6 Silver staining and mass spectrometry (MS)

The Flag‐RNF12 and Flag‐Vector plasmids were used to transiently transfect HEK293 cells, which were subsequently collected and resuspended in IP lysis buffer containing proteinase inhibitor. The lysates were used for immunoblotting and co‐IP, followed by silver staining and MS. Silver staining was conducted as follows. A solution containing 30% ethanol and 10% glacial acetic acid was used to fix electrophoresis for 30 min to 3 h. After fixing, 10% ethanol solution to wash electrophoresis twice, 5–10 min each time. Then, silver stain sensitize and silver stain to soak electrophoresis and deionized water was used to wash it. Next, silver stain developer was used to soak electrophoresis and deionized water was used to wash it. Finally, silver stain stop buffer was used to soak electrophoresis and stop development. After completing silver staining, electrophoresis could be stored in 2% acetic acid, which is subsequently delivered to the company to conduct MS analysis.

### 2.7 Immunofluorescence

Cells were cultured on Petri dishes for confocal microscopy. After aspirating the culture medium, the cells were fixed in 4% paraformaldehyde for 15 min at room temperature and then permeabilized using 0.5% Triton X‐100 for 20 min. After blocking with 3% BSA for 1 h, the cells were incubated with the specific primary antibody at 4°C overnight. On the second day, the cells were washed three times with PBS and incubated with the corresponding secondary antibody for 1 h in a humidified box at room temperature. Finally, DAPI (Sigma‐Aldrich) was used to counter‐stain the nuclei for 5 min in the dark.

### 2.8 Flow cytometry

APC anti‐human EGFR Antibody (352905, BioLegend) was used for flow cytometry. The cells were collected in a tube and washed three times with PBS. Then, the cells were incubated with the fluorescently labelled antibody at room temperature for 30 min in the dark. After incubation, the antibody solution was removed by centrifuging the cells at 200 ***g*** and re‐suspending them in PBS. Finally, the cells were analysed on a flow cytometer (BD Bioscience).

### 2.9 Cell proliferation and colony formation assays

After digesting and re‐suspending the cells, 1000 cells were added into the 96‐well plate and placed back in the cell incubator for culturing. Next, the cells in the 96‐well plate were incubated with CCK8 solution for 1–2 h at a fixed time every day. And the OD value was measured by the microplate reader. Meantime, the same number of cells was added into the 6‐well plate and cultured them for 14 days. Finally, paraformaldehyde was used to fix the cells and crystal violet was used to stain the cells.

### 2.10 Transwell migration and invasion assay

A transwell plate containing 8‐μm pores (Corning) was used for the migration and invasion assays. For the migration assay, 200 μL of serum‐free DMEM containing 1 × 10^5^ cells was placed into the upper chamber, and 650 μL DMEM with 10% FBS was placed into the lower chamber. For the invasion assay, the chamber inserts were pre‐coated with 2 mg/mL Matrigel (BD Biosciences) overnight. Then, 200 μL of serum‐free DMEM containing 5 × 10^5^ cells was placed into the upper chamber. After culturing for 24 h, cells in the lower chamber were fixed using 4% neutral formalin in PBS for 15 min and stained with crystal violet for 15 min. Then, the cells in each field were counted using bright‐field microscopy (DM400B, Leica Corporation) to evaluate the migration and invasion ability.

### 2.11 Wound‐healing assay

Cells in 6‐well plates were allowed to grow to between 95% and 100% confluence, after which a 200 μL pipette tip was used to scratch the cell monolayer cells to form a wound. Phase contrast microscopy (DM400B, Leica Corporation) was used to record the process of cells migrating to fill the scratch for 24 h. Photographs of wound closure were used for analysis of migration ability.

### 2.12 Animal experiments

All animal experiments were conducted in accordance with the National Institutes of Health guidelines (NIH publication 86‐23, revised 1985), and were approved by the Committee on the Ethics of Animal Experiments of Tongji Medical College, HUST. Animal experiments including the xenograft model, orthotopic implantation model and mouse pulmonary metastasis model were conducted as described previously.^15^, ^16^ All male Balb/c athymic nude mice are 6 weeks old and raised on specific‐pathogen‐free conditions. For the xenograft tumour model, suspending 1 × 10^6^ indicated tumour cells with 100 μL of serum‐free DMEM and inoculating subcutaneously them into the flanks of 6‐week‐old nude mice. Then all experimental mice were raised for 30 days and sacrificed to measure the weight of subcutaneous tumours. For mouse pulmonary metastasis, suspending 1 × 10^6^ indicated tumour cells with 100 μL of serum‐free DMEM and injecting them into the tail vein of nude mice. Then all experimental mice were raised for 30 days and sacrificed to measure the metastatic foci in the lung. For orthotopic implantation model, suspending 1 × 10^6^ indicated tumour cells with 30 μL of serum‐free DMEM and inoculating them into the liver of nude mice. Then all experimental mice were raised for 30 days and sacrificed to measure weight of tumours.

### 2.13 Statistical analysis

SPSS 22.0 (IBM Corp.). and GraphPad prism 8 software (GraphPad Software, Inc.) were used for all statistical analyses. The chi‐squared test was used to assess the statistical significance of the relationship between RNF12 levels and clinicopathological features. Kaplan–Meier survival analysis and two‐tailed Student's *t*‐test were used to assess the differences of survival rates. Differences with *p* < 0.05 were defined as statistically significant.

## 3 RESULTS

### 3.1 High RNF12 expression was correlated with a poor prognosis

**FIGURE 1:**
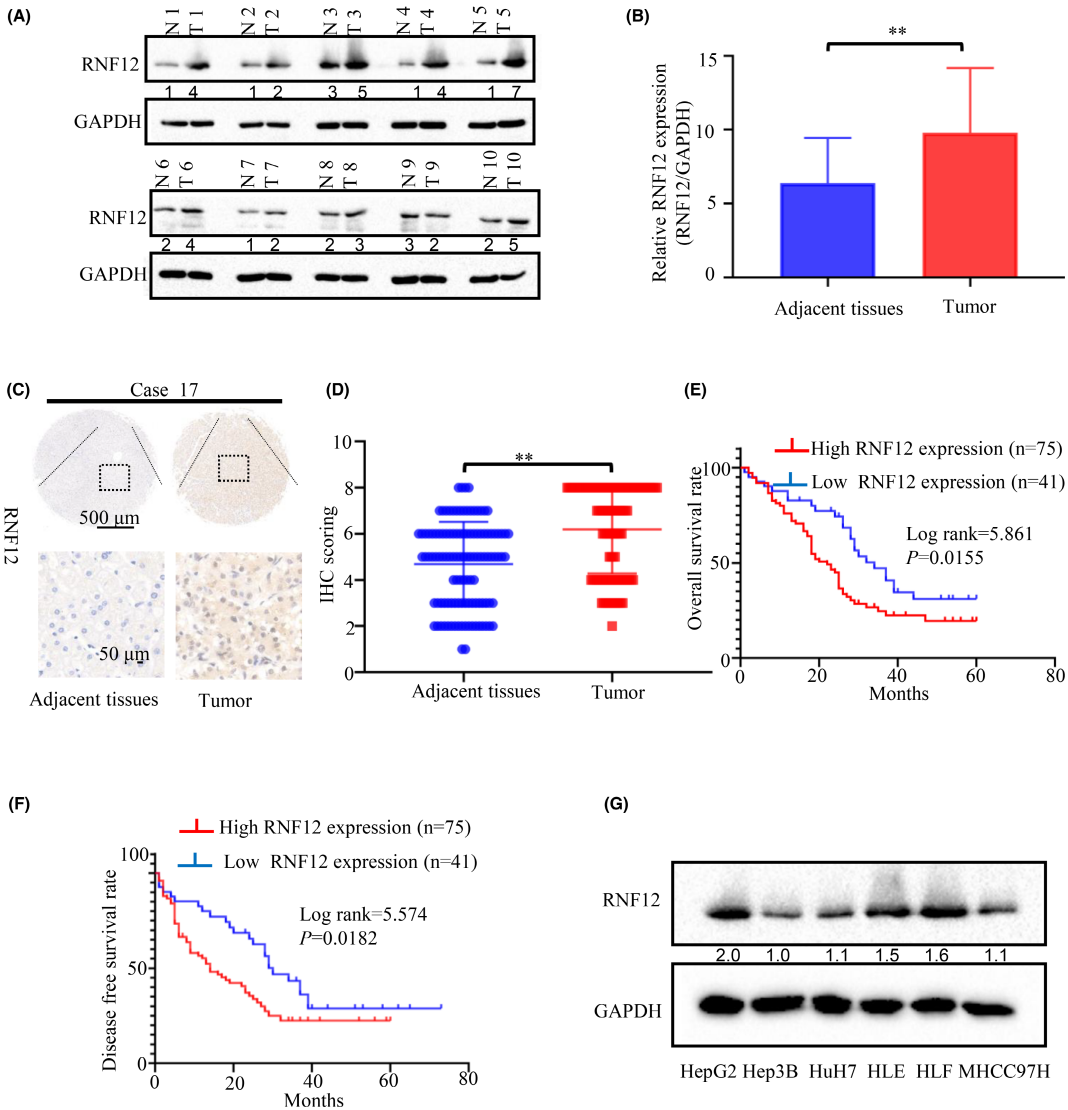
High RNF12 expression was found in liver cancer, in correlation with worse clinicopathological features and a poor prognosis. (A) The protein levels of RNF12 were compared in 20 paired liver cancer samples and their corresponding adjacent normal tissues by western blot analysis. (B) Statistical analysis confirmed a significant increase of RNF12 levels in liver cancer tissues compared with paired adjacent non‐cancerous liver tissues (**p < 0.01, GAPDH was used as the internal control). (C) Two representative images of immunohistochemical staining for RNF12 in 116 pairs of liver cancer tissues and adjacent normal tissues were shown. (D) Statistical analysis of the immunohistochemistry results. (E) Kaplan–Meier overall survival curves of two liver cancer groups: high RNF12 group: *n* = 75; low RNF12 group: *n* = 41. (F) Kaplan–Meier disease‐free survival curves of two liver cancer groups: high RNF12 group: *n* = 75; low RNF12 group: *n* = 41. (G) The protein levels of RNF12 in a panel of HCC cell lines and normal liver tissue by western blot analysis.

RNF12 has been reported to be dysregulated in some tumours, such as glioblastoma and breast cancer.^12^, ^13^ However, the roles of RNF12 in liver cancer have not been explored in detail. In order to explore the roles of RNF12 in the progression of liver cancer, we first used GEPIA to analyse the expression and prognostic value of RNF12 in liver from TCGA.^17^ Compared to normal liver tissues, the results showed RNF12 was overexpressed in liver cancer specimens (Figure S1A). The results of TCGA showed that patients with high RNF12 expression also had shorter OS (*p* = 0.034; Figure S1B) and DFS (*p* = 0.037; Figure S1C). We used western blotting to detect the expression of RNF12 in 20 paired human liver cancer and adjacent normal tissue samples. The results showed that the expression of RNF12 in tumour samples was higher than in adjacent tissues (Figures 1A,B). Additionally, we detected the expression of RNF12 in 116 paired liver cancer and adjacent normal tissues using immunohistochemistry (IHC). The results of IHC demonstrated that RNF12 was overexpressed in liver cancer specimens compared to the corresponding adjacent tissues (*p* < 0.001; Figure 1C,D). To further explore the relationship of the expression of RNF12 with clinicopathological features and prognosis, we divided patients into low and high expression groups based on the immunostaining score. The results demonstrated that high expression of RNF12 was related to tumour size (*p* = 0.03), tumour differentiation (*p* = 0.02), tumour stage (BCLC stage *p* = 0.025 and TNM stage *p* = 0.017) and tumour recurrence (*p* = 0.012) (Table 1). Next, Kaplan–Meier survival analysis was used to explore the relationship between the expression of RNF12 and OS or DFS. The results showed that patients with high RNF12 expression had shorter OS (*p* = 0.0155; Figure 1E) and DFS (*p* = 0.0182; Figure 1F). At the same time, we also used western blotting to detect the expression of RNF12 in liver cancer cell lines (Figure 1G).

**TABLE 1:** The association between RNF12 expression and clinical characteristics in 116 patients with primary HCC.

		RNF12 expression		
Features	Total	High expression	Low expression	*p*‐Value
Sex				
Male	95	63	32	0.426
Female	21	12	9	
Age (years)				
≤50	52	35	19	0.795
>50	64	40	24	
ALT				
<70	106	70	36	0.311
≥70	10	5	5	
AST				
<70	101	66	35	0.686
≥70	15	9	6	
Serum AFP (ng/mL)				
<400	62	38	24	0.417
≥400	54	37	17	
Tumour size (cm)*				
>5	77	57	20	0.03
≤5	39	18	21	
Tumour number				
Single	90	57	33	0.580
Multiple	26	18	8	
BCLC stage				
0 + A	75	54	21	0.025
B + C	41	21	20	
TNM stage				
I + II	86	61	25	0.017
III + IV	30	14	16	
Differentiation				
Well/moderate	73	53	20	0.020
Poor	43	22	21	
Vascular invasion				
Yes	23	17	6	0.3
No	93	58	35	
Tumour capsule				
Absent	55	36	19	0.864
Present	61	39	22	
Recurrence				
Yes	58	44	14	0.012
No	58	31	27	

### 3.2 RNF12 promoted growth of liver cancer in vitro and in vivo

**FIGURE 2:**
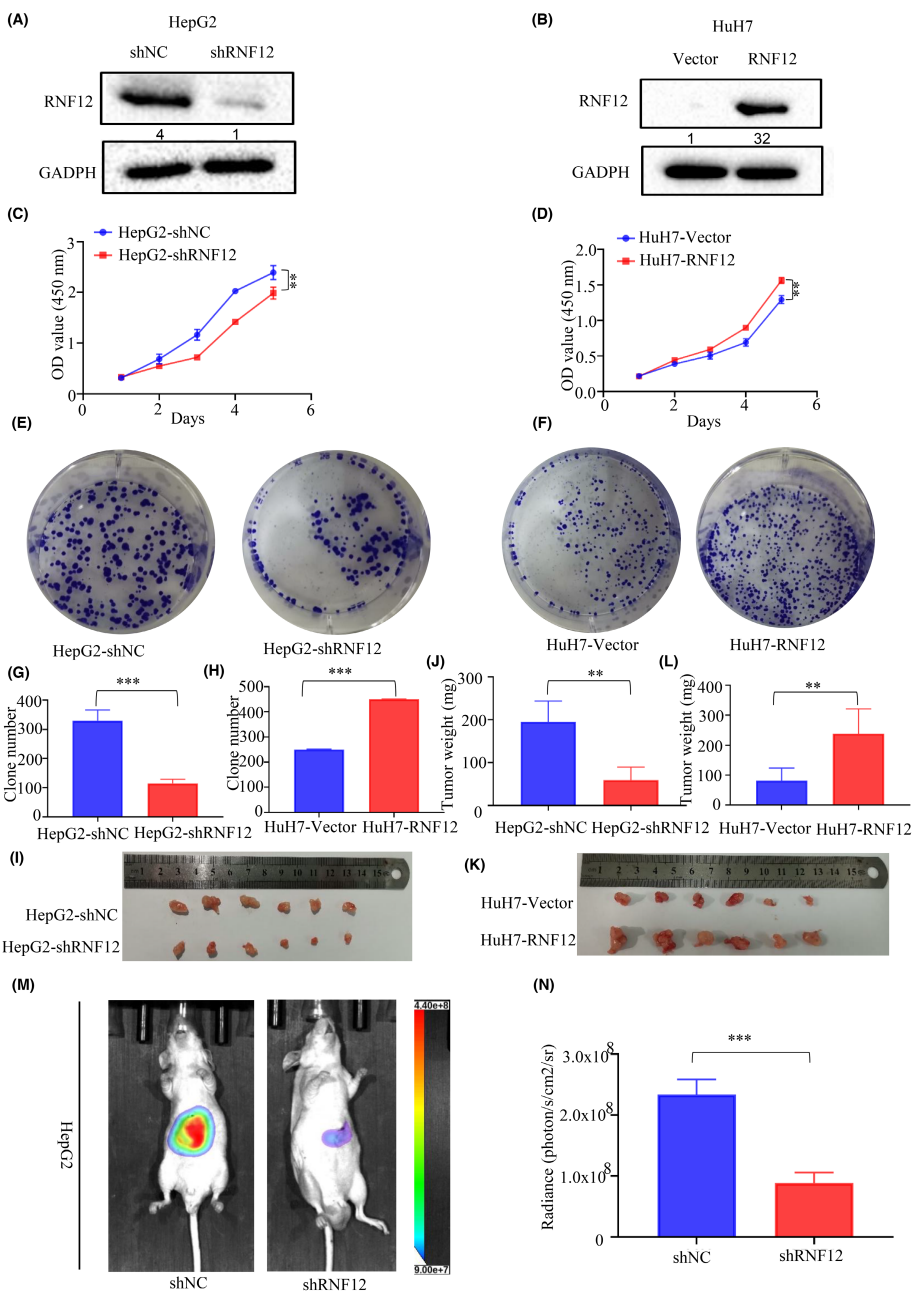
RNF12 promoted the tumour growth of liver cancer in vitro and in vivo. (A, B) Western blots confirming that RNF12 was successfully knocked down in HepG2 cells and overexpressed in Huh7 cells. (C, D) CCK8 assay showing that RNF12 knockdown restrained proliferation in HepG2 cells, and RNF12 overexpression promoted proliferation in Huh7 cells (*n* = 6). (E, G) Colony formation assay showing that RNF12 knockdown restrained proliferation in HepG2 cells. (F, H) Colony formation assay showing that RNF12 overexpression promoted proliferation in Huh7 cells (*n* = 3). (I, J) Xenograft model showing that RNF12 knockdown restrained tumour growth in vivo. (K, L) Xenograft model showing that RNF12 overexpression promoted tumour growth in vivo. (M, N) Orthotopic implantation model (*n* = 6 for each group) showing that the growth of HepG2 cells with RNF12 knockdown was weaker than that of control cells. Data are presented as mean ± SD, **p < 0.01, ***p < 0.001.

In 2000 and 2011, Dr Robert Weinberg and Dr Douglas Hanahan proposed 10 hallmarks of cancer, among which sustained proliferation and invasion and metastasis are two of hallmarks of cancer.^18^ Therefore, the study of cell proliferation, invasion and metastasis is the basis of the study of tumour development. Since high RNF12 expression was found in liver cancer and was correlated with worse outcomes, RNF12 had a likely tumour promoter role in liver cancer. Consequently, we decided to explore the effect of RNF12 in the growth and metastasis of liver cancer. First, we infected HepG2 and Huh7 cells with lentiviral vectors to obtain stable cell lines with RNF12 overexpression or knockdown. Then, the overexpression of RNF12 in Huh7 cells and the RNF12 knockdown in HepG2 cells was confirmed by western blot analysis (Figure 2A,B). Then we used CCK8, colony formation, xenograft model and orthotopic implantation model to explore the effect of RNF12 in the growth of liver cancer. The results of the CCK8 assay showed that the growth of HepG2 with knockdown of RNF12 was slower than that of control cells (Figure 2C). Conversely, the growth of Huh7 cells overexpressing RNF12 was faster than that of control cells (Figure 2D). Similarly, the results of colony formation assays demonstrated that the clonogenicity of HepG2 cells with knockdown of RNF12 was lower than that of control cells (Figure 2E,G). Conversely, the clonogenicity of Huh7 cells overexpressing RNF12 was increased compared to control cells (Figure 2F,H). Next, we used a xenograft model and an orthotopic implantation model to explore the effect of RNF12 on tumour growth in vivo. The xenograft model showed that the tumour size of HepG2 cells with RNF12 knockdown was smaller than that of control cells (Figure 2I,J). By contrast, the tumour size of Huh7 cells with RNF12 overexpression was larger than that of control cells (Figure 2K,L). Representative photographs of haematoxylin and eosin staining of the tumours are shown in Figure S2A,B. Furthermore, the orthotopic implantation model showed that the growth ability of HepG2 cells with RNF12 knockdown was weaker than that of control cells (Figure 2M,N), and representative photographs of haematoxylin and eosin staining are shown in Figure S2C. Taken together, these results showed that RNF12 could promote tumour growth of liver cancer in vitro and in vivo.

### 3.3 RNF12 promoted invasion and migration of liver cancer in vitro and in vivo

**FIGURE 3:**
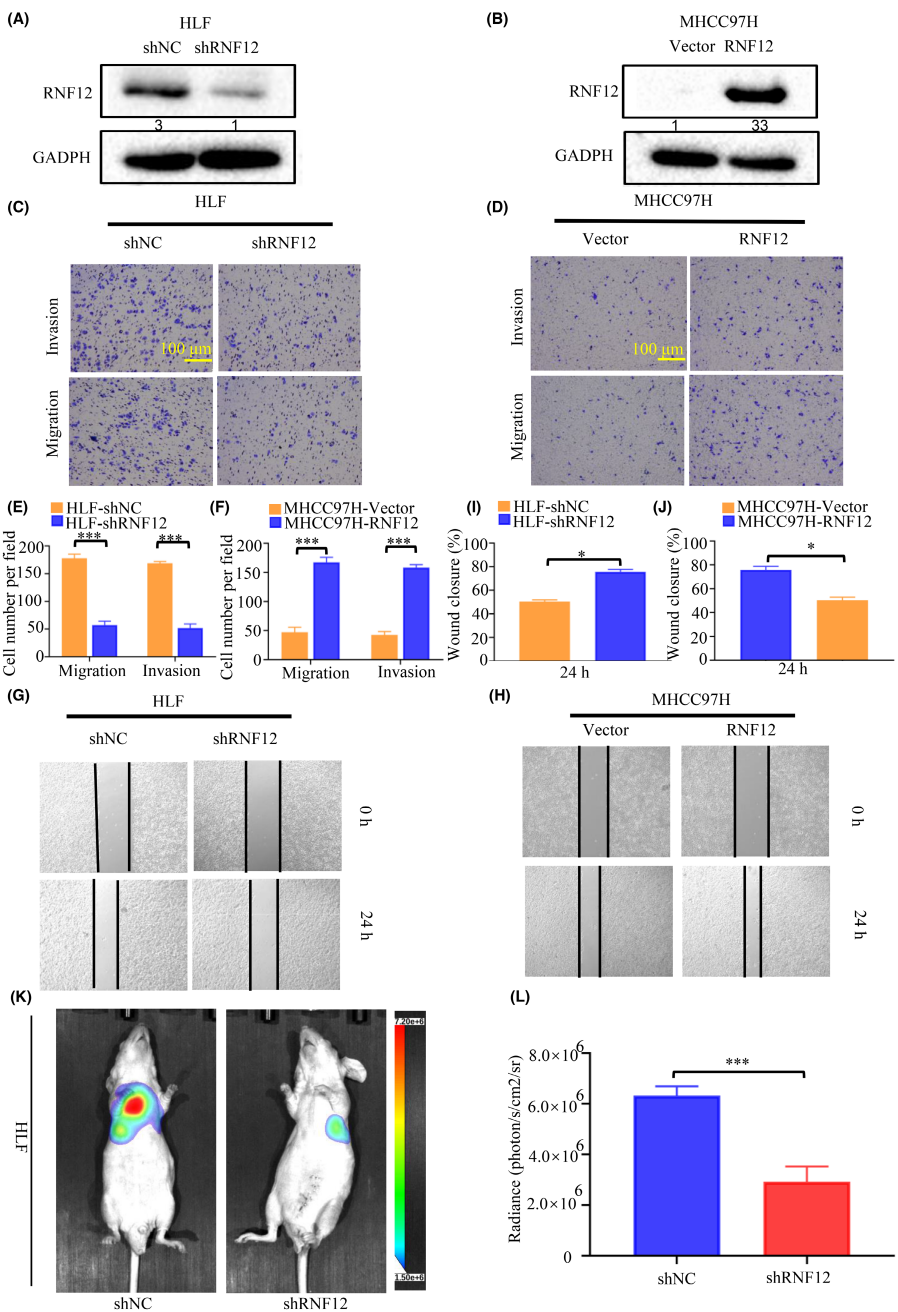
RNF12 promoted tumour metastasis of liver cancer in vitro and in vivo. (A, B) Western blots showing that RNF12 was successfully knocked down in HLF cells and overexpressed in MlHCC97H cells. (C, E) Transwell assay showing that RNF12 knockdown restrained metastasis in HLF cells (*n* = 3). (D, F) Transwell assay showing that RNF12 overexpression promoted metastasis in MHCC97H cells (*n* = 3). (G, I) Wound healing assay showing that RNF12 knockdown restrained metastasis in HLF cells (*n* = 3). (H, J) Wound healing assay showing that RNF12 overexpression promoted metastasis in MHCC97H cells (*n* = 3). (K, L) Mouse pulmonary metastasis models (*n* = 6 for each group). showing that the metastatic potential of HLF cells with RNF12 knockdown was weaker than that of control cells. Data are presented as mean ± SD. *p* < 0.05, ***p < 0.001.

After exploring the effect of RNF12 on growth of liver cancer, we further explored the effect of RNF12 on the invasion and migration liver cancer. First, we obtained stable cell lines (MHCC97H and HLF) with RNF12 overexpression or knockdown to evaluate the effect of RNF12 on tumour metastasis. The overexpression of RNF12 in MHCC97H and RNF12 knockdown in HLF cells was confirmed by western blot analysis (Figure 3A,B). Then the in vitro metastatic potential of the resulting cell lines was assessed using transwell migration and invasion assays, as well as the cell monolayer‐based scratch wound healing assay. The results of transwell migration and invasion assays showed that the mobility of HLF cells with the knockdown of RNF12 was reduced compared to control cells (Figure 3C,E). Conversely, more MHCC97H cells with RNF12 overexpression migrated than did control cells (Figure 3D,F). The results of the wound healing assay showed the wound closure of HLF with RNF12 knockdown was faster than with control cells (Figure 3G,I). Conversely, the wound closure of MHCC97H with RNF12 overexpression was slower than in control cells (Figure 3H,J). Next, we used the mouse pulmonary metastasis model to further explore the role of RNF12. The results showed that the metastatic potential of HLF cells with RNF12 knockdown was weaker than that of control cells (Figure 2K,L). Accordingly, the number and size of metastatic lung nodules of HLF cells with RNF12 knockdown was smaller than that of control cells (Figure S3A,B). Taken together, these results indicated that RNF12 could promote tumour metastasis of liver cancer in vitro and in vivo.

### 3.4 RNF12 interacted with EGFR via its central region

**FIGURE 4:**
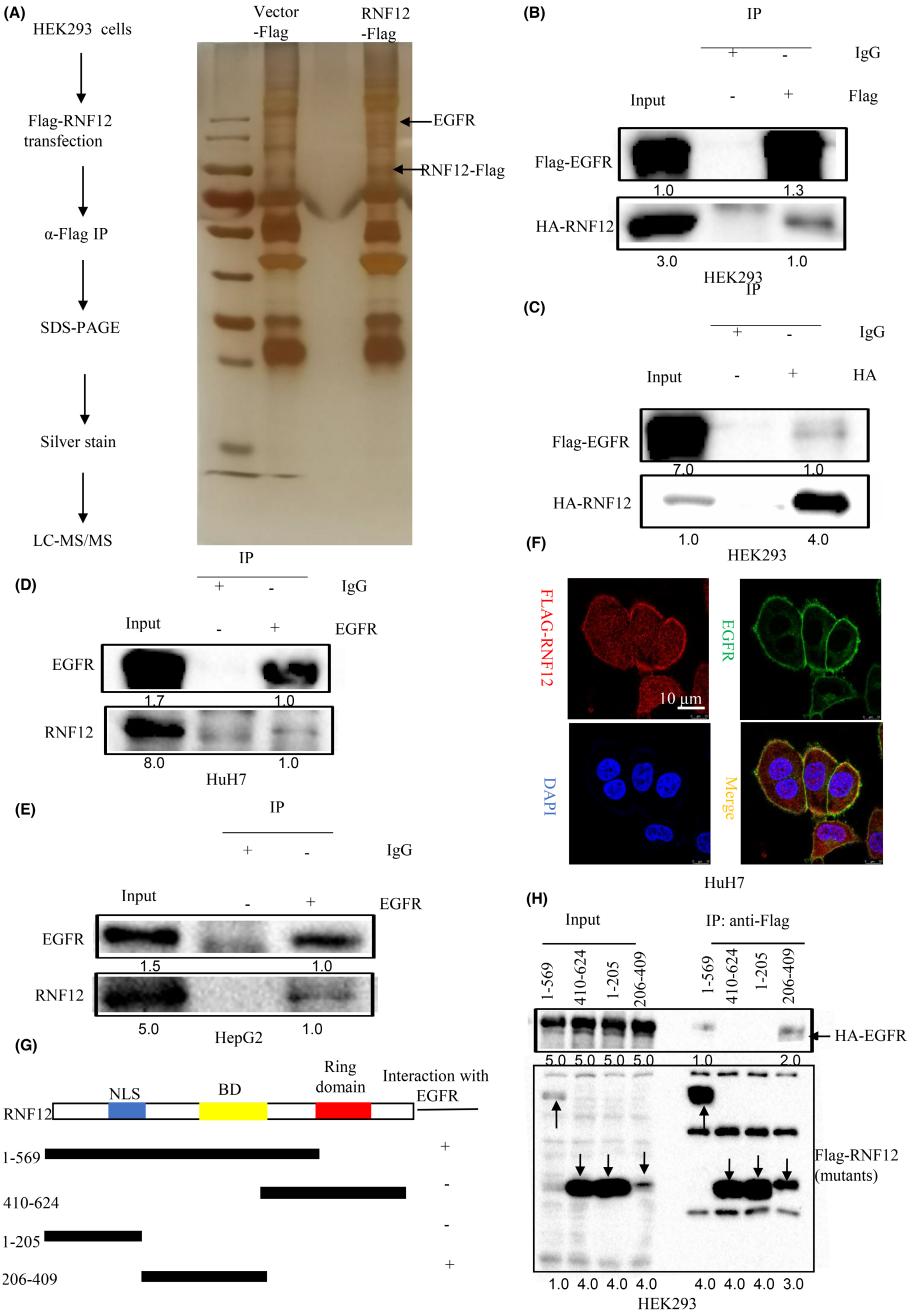
Identification of EGFR as a binding partner of RNF12. (A) HEK293 cells overexpressing FLAG‐tag or FLAG‐tagged RNF12 were subjected to immunoprecipitation and analysed by gradient elution LC–MS/MS. (B, C) RNF12 could interact with exogenous EGFR. (D, E) RNF12 could interact with endogenous EGFR. (F) Confocal immunofluorescence showing that RNF12 and EGFR co‐localized in the cell membrane of Huh7 cells. (G) Construction of a series of RNF12 truncation mutants. (H) The central region (aa 206–409) of RNF12 mediates its interaction with EGFR.

As the above results showed that RNF12 could promote the growth and migration of liver cancer to contribute to the progression of liver cancer, we then explore the mechanisms by which RNF12 plays the above roles. Based on the existing literature, RNF12 mainly exerts its function by binding to other proteins. Hence, a combined IP/MS approach was used to identify novel protein–protein interactions of RNF12 to explore the underlying mechanisms of the effect of RNF12 on the tumorigenesis of liver cancer. HEK293 cells overexpressing FLAG‐tag or FLAG‐tagged RNF12 were subjected to immunoprecipitation and gradient elution LC–MS/MS (Figure 4A). The newly identified interaction partners of RNF12 included EGFR (Tables [Link], [Link]). Notably, dysregulated EGF/EGFR signal activation is commonly found in cancers.^19^, ^20^ Whether RNF12 could activate EGF/EGFR signalling by interacting with EGFR to promote the progression of liver cancer was therefore a pertinent question. To further confirm the MS data, exogenous co‐IP of RNF12 and EGFR was performed in HEK293T cells. The results showed that RNF12 could interact with exogenous EGFR (Figure 4B,C). Furthermore, endogenous co‐IP was conducted in Huh7 and HepG2 cells to confirm their endogenous interaction. As expected, RNF12 was also found to interact with endogenous EGFR (Figure 4D,E). Additionally, confocal immunofluorescence microscopy was used to assess the co‐localization of RNF12 and EGFR in liver cancer cell lines. The results showed RNF12 and EGFR had a high degree of spatial concordance in Huh7 cells (Figure 4F). To identify the EGFR‐binding domain of RNF12, we constructed a series of RNF12 truncation mutants (Figure 4G). The results showed that Both RNF12 (aa 1–569) and RNF12 (aa 206–409) strongly interact with EGFR, whereas RNF12 (aa 1–205) and RNF12 (aa 410–624) do not (Figure 4H). These results indicated that the central region of RNF12 (aa 206–409) mediates its interaction with EGFR.

### 3.5 RNF12 decreased the internalization of EGFR and activated AKT signalling pathway

**FIGURE 5:**
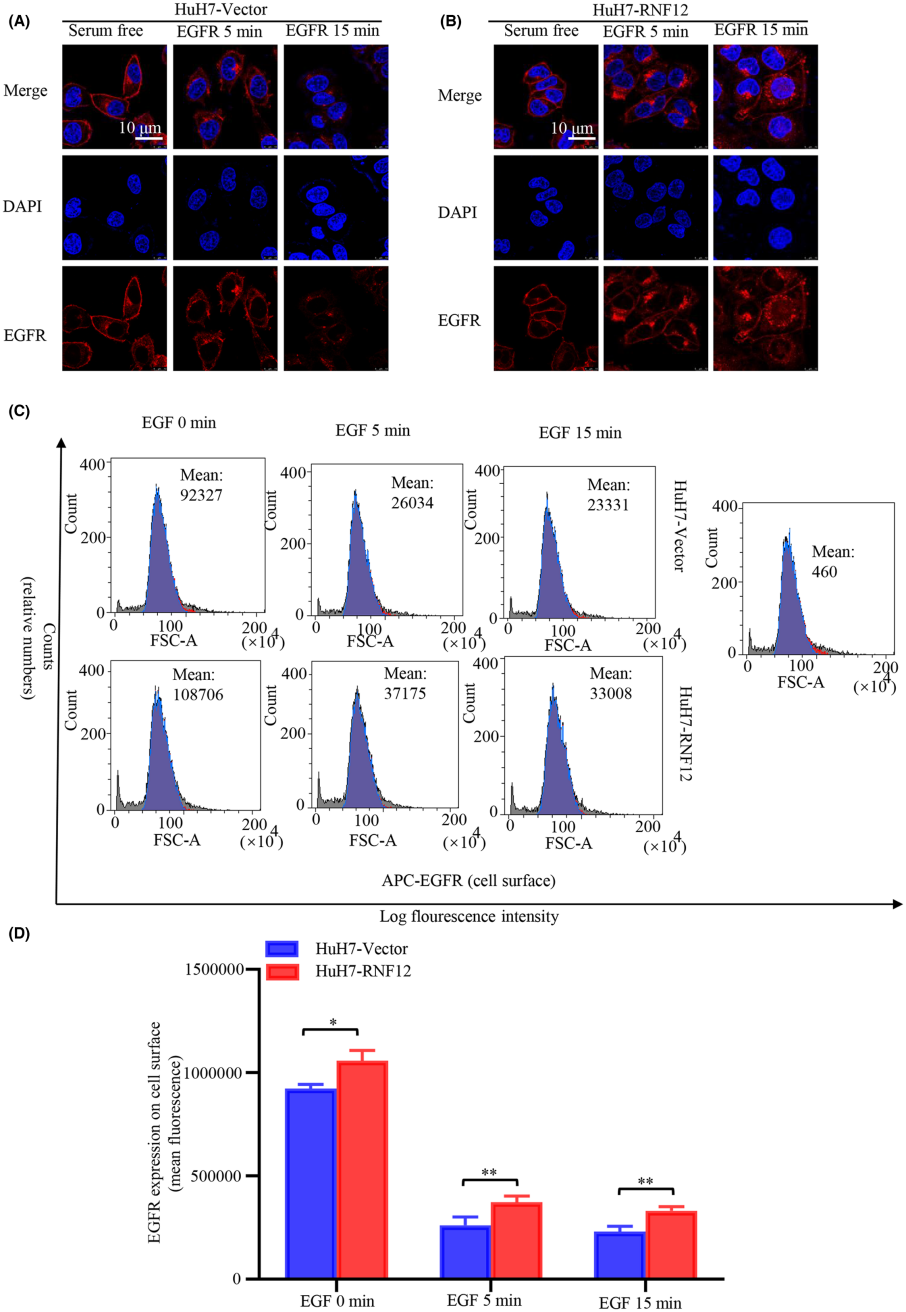
RNF12 decreased the internalization of EGFR. (A, B) Confocal immunofluorescence imaging showing that the internalization of EGFR following EGF treatment was slower in Huh7 cells overexpressing RNF12 than in control cells. (C, D) Fluorescence activated cell sorting (FACS) analysis showing that the EGFR levels on the surface of Huh7 cells overexpressing RNF12 were higher than in control cells (*n* = 3). Data are presented as mean ± SD. *p < 0.05, **p < 0.01.

**FIGURE 6:**
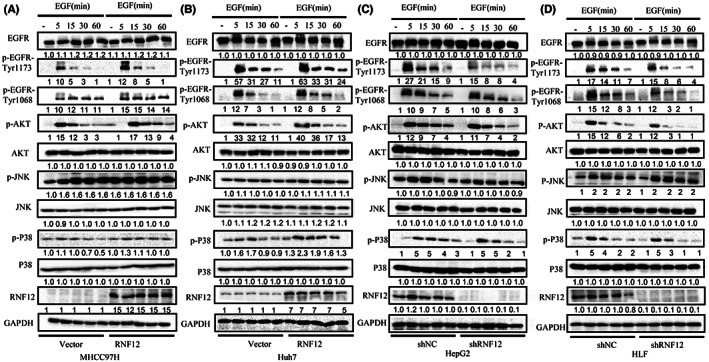
RNF12 activates AKT signalling pathway. (A, B) Western blots showing that RNF12 overexpression increased the phosphorylation of EGFR and its downstream signalling molecules in MHCC97H and Huh7 cells. (C, D) Western blots showing that RNF12 knockdown decreased the phosphorylation of EGFR and its downstream signalling molecules in HepG2 and HLF cells.

As RNF12 could interact with EGFR, we then explored the effect of RNF12 on EGFR and its downstream signalling. The intracellular localization of EGFR tightly controls its signalling activity. Epidermal growth factor (EGF) interacts with EGFR and activates receptor signalling, which triggers a complex cascade, which in turn leads to receptor inactivation by promoting its endocytosis, lysosomal targeting and degradation.^21^, ^22^ Since the results of immunofluorescence confocal microscopy showed that RNF12 and EGFR were co‐localized in the cell membrane, we also investigated the effect of RNF12 on EGF‐induced endocytosis. EGF was used to stimulate Huh7 with RNF12 overexpression or control cells followed by confocal immunofluorescence imaging and fluorescence activated cell sorting (FACS). The results of confocal immunofluorescence imaging showed that the internalization of EGFR following EGF treatment was slower in Huh7 cells with RNF12 overexpression than in control cells (Figure 5A,B). Accordingly, the results of FACS analysis showed that the EGFR levels on the surface of Huh7 cells with RNF12 overexpression were higher than in the control cells (Figure 5C,D). Taken together, these results indicated that RNF12 could decrease the internalization of EGFR. Consequently, we further explored the effect of RNF12 on the EGF/EGFR signalling pathway using western blotting to investigate the changes of EGFR phosphorylation and its downstream signalling under EGF stimulation. The results of western blot analysis demonstrated that the phosphorylation levels of EGFR Thy‐1068 and Thy‐1173 were higher in RNF12‐overexpressing cells than in control cells (Figure 6A,B). Conversely, the phosphorylation levels at both residues were lower in RNF12 knockdown cells than in control cells (Figure 6C,D). JNK signalling, p38‐MAPK signalling and PI3K‐AKT signalling are classical downstream signalling pathways of EGFR that play important roles in cancer progression.^7^, ^23^, ^24^ Accordingly, we also investigated the effects of RNF12 on these pathways and found that the phosphorylation levels of AKT and P38 were higher in RNF12‐overexpressing cells and lower in RNF12 knockdown cells compared to control cells (Figures 6A–D). However, the change in the phosphorylation level of AKT was more pronounced.

### 3.6 RNF12 promoted the proliferation and migration of liver cancer via AKT signalling

**FIGURE 7:**
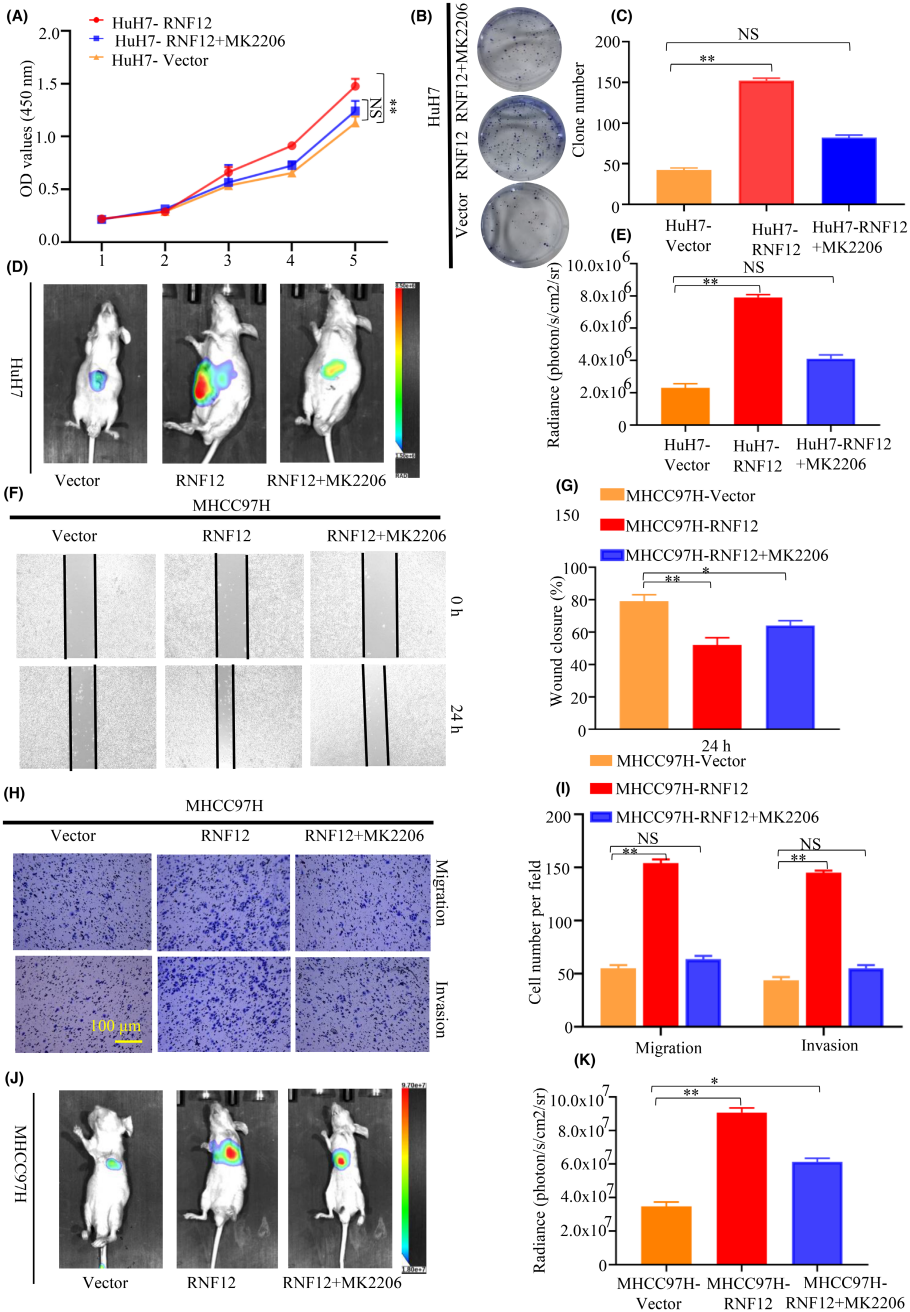
AKT inhibitor attenuated RNF12‐mediated effect in liver cancer in vitro and in vivo. (A–E) The results of CCK8 (*n* = 6), colony formation (*n* = 3) and orthotopic implantation model (*n* = 6 for each group) showed that the specific AKT inhibitor MK2206 blocked the induction of cell proliferation by RNF12 overexpression in vitro and in vivo. (F–K) The results of Transwell (*n* = 3), wound healing assay (*n* = 3) and mouse pulmonary metastasis models (*n* = 6 for each group) showed that the specific AKT inhibitor MK2206 blocked the upregulation of cell migration by RNF12 overexpression in vitro and in vivo. Data are presented as mean ± SD, *p < 0.05, **p < 0.01, NS, no significance.

Since the results of this study indicated that RNF12 markedly increased the phosphorylation of AKT in liver cancer cell lines and AKT signalling plays an important role in the proliferation and migration of liver cancer,^25^ we explored the effects of AKT signalling on the regulation of liver cancer cell proliferation and migration by RNF12. The effect of RNF12 regulation of liver cancer cell proliferation was detected in Huh7 cells with or without AKT signalling blockade (Figure S4A). The CCK8 and colony formation assays showed that specific shRNA‐mediated knockdown of AKT gene expression could reverse the induction of cell proliferation by RNF12 overexpression (Figure S4C–E). As a specific AKT inhibitor, MK2206 can effectively inhibit AKT signalling. The CCK8 and colony formation assays showed that MK2206 could block the induction of cell proliferation following RNF12 overexpression (Figure 7A–C). Meantime, the orthotopic implantation model showed that MK2206 could block the induction of cell proliferation following RNF12 overexpression (Figure 7D,E). These results showed that AKT signalling is involved in the regulation of liver cancer cell proliferation by RNF12 in vitro and in vivo. Similarly, the effect of RNF12 on liver cancer cell migration was detected in MHCC97H lines with or without AKT signal blockade (Figure S4B). The transwell migration and invasion assays demonstrated that specific shRNA‐mediated knockdown of AKT gene expression could block the induction of cell migration by RNF12 overexpression (Figure S4F–I). Additionally, the transwell migration and invasion assays demonstrated that MK2206 also blocked the induction of cell migration by RNF12 overexpression (Figures 7F–I). Meantime, the mouse pulmonary metastasis model showed that MK2206 could block the induction of cell migration by RNF12 overexpression (Figure 7J,K). The data showed that AKT signalling are involved in the regulation of liver cancer cell proliferation and migration by RNF12 in vitro and in vivo.

## 4 DISCUSSION

Ring finger protein family function as an E3 ubiquitin ligase and a co‐transcription factor, which has been reported to be involved in cancer progression and the regulation of signalling pathways. For example, RNF126 could ubiquitinate PTEN and induce its proteasome degradation, thereby regulating the PI3K‐AKT signalling pathway to promote the cellular proliferation and migration of bladder cancer.^26^ In lung adenocarcinoma, RNF115 could ubiquitinate APC and result in its degradation, thus regulating Wnt/β‐Catenin signalling to promote cellular proliferation.^27^ Meanwhile, RNF128 could promote EGFR phosphorylation by interacting with p53, thereby regulating MAPK/ MMP‐2 signalling pathway to the invasion and metastasis of oesophageal squamous cell carcinoma.^28^ C‐CBL, also as a RING finger E3 ubiquitin ligase, could ubiquitinate EGFR and promote its internalization and degradation.^29^

RNF12, as a member of the ring finger protein family, has been reported to be dysregulated in many malignancies.^12^, ^13^, ^30^ RNF12 was reported to be overexpressed in glioblastoma, which was correlated with a poor prognosis.^13^ Furthermore, RNF12 was found to interact with and target RB1 for degradation to stimulate the MAPK pathway and promote the malignant proliferation of glioblastoma.^13^ In addition, high RNF12 expression was found in breast cancer and was associated with a poor prognosis.^12^ RNF12 was also found to interact with and target Smad7 for degradation to promote TGF‐β‐driven metastasis in breast cancer.^12^ In this study, we found that RNF12 was highly expressed in liver cancer. At the same time, high RNF12 expression in liver cancer was correlated with a poor prognosis and worse clinicopathological features. Furthermore, RNF12 knockdown attenuated and RNF12 overexpression increased liver cancer proliferation, motility, and invasiveness in vitro and in vivo. These data further indicated that RNF12 exerts a tumour promoter role and might become a potential therapeutic target in liver cancer.

EGFR exerts a vital role in normal cellular growth and differentiation, and its disorder is found in the pathogenesis of many cancers.^31^ Endocytic downregulation plays an important role in terminating EGFR signalling after ligand stimulation, and EGF‐induced EGFR internalization is a vital link in endocytic downregulation.^32^ Specifically speaking, EGFR undergoes dimerization and self‐phosphorylation of tyrosine residues under ligand stimulation, which leads to EGFR activation. Activated EGFR rapidly internalizes and targets early endosomes. Some of the EGFR in early endosomes was recycled back to the plasma membrane.^33^ The others were transferred to lysosomes, which caused the degradation of EGFR and signalling termination.^33^ In this study, we found that RNF12 could decrease EGF‐induced EGFR internalization and increase the level of EGFR phosphorylation. However, the exact mechanism is not clear. Many proteins are involved in the regulation of EGFR internalization, such as Grb2 and c‐CBL.^32^ For example, Grb2 could interact with and activate EGFR, which stimulates EGFR internalization and degradation. The possible interaction of RNF12 with these proteins to decrease EGF‐induced EGFR internalization needs further investigation in future studies.

In this study, we only focused on and verified the interaction between RNF12 and EGFR. Besides, the results of IP‐MS also showed many other interactions of RNF12. In the different proteins of IP‐MS (Table S4), there are many transcription‐related proteins, such as EEF1A1 and HNRNPC. EEF1A1 is an isoform of the alpha subunit of the elongation factor‐1 complex and is responsible for the enzymatic delivery of aminoacyl tRNAs to the ribosome.^34^ RNF12 might interact with EEF1A1 and regulate the enzymatic delivery of aminoacyl tRNAs to the ribosome, which promotes the progression of liver cancer. HNRNPC is an RNA binding protein, which is responsible for pre‐mRNAs in the nucleus and influences pre‐mRNA processing.^35^ RNF12 might interact with HNRNPC to regulate the process of pre‐mRNAs in the nucleus, thus promoting the progression of liver cancer. Meantime, the different proteins of IP‐MS (Table S4) contained PPP1CC, which was a serine/threonine phosphatase and regulated the activation of many signalling pathways.^36^ RNF12 might interact with PPP1CC to promote the progression of liver cancer by regulating the signalling pathway.

Abnormal expression of components of growth factor receptors that control cell growth and survival is often involved in cancer progression.^6^ The PI3K‐AKT signalling pathway is an important intracellular pathway regulating cell growth, survival, cellular metabolism and cytoskeletal reorganization of cells in response to a range of signals.^37^, ^38^ Furthermore, the PI3K‐AKT signalling pathway is dysregulated in various cancers, including liver cancer, and is involved in cancer progression.^39^ In this study, we found that RNF12 could activate the PI3K‐AKT signalling pathway. RNA silencing and pharmaceutical inhibition experiments confirmed that activation of the PI3K‐AKT signalling pathway is required for the oncogenic effects of RNF12 in liver cancer.

**FIGURE 8:**
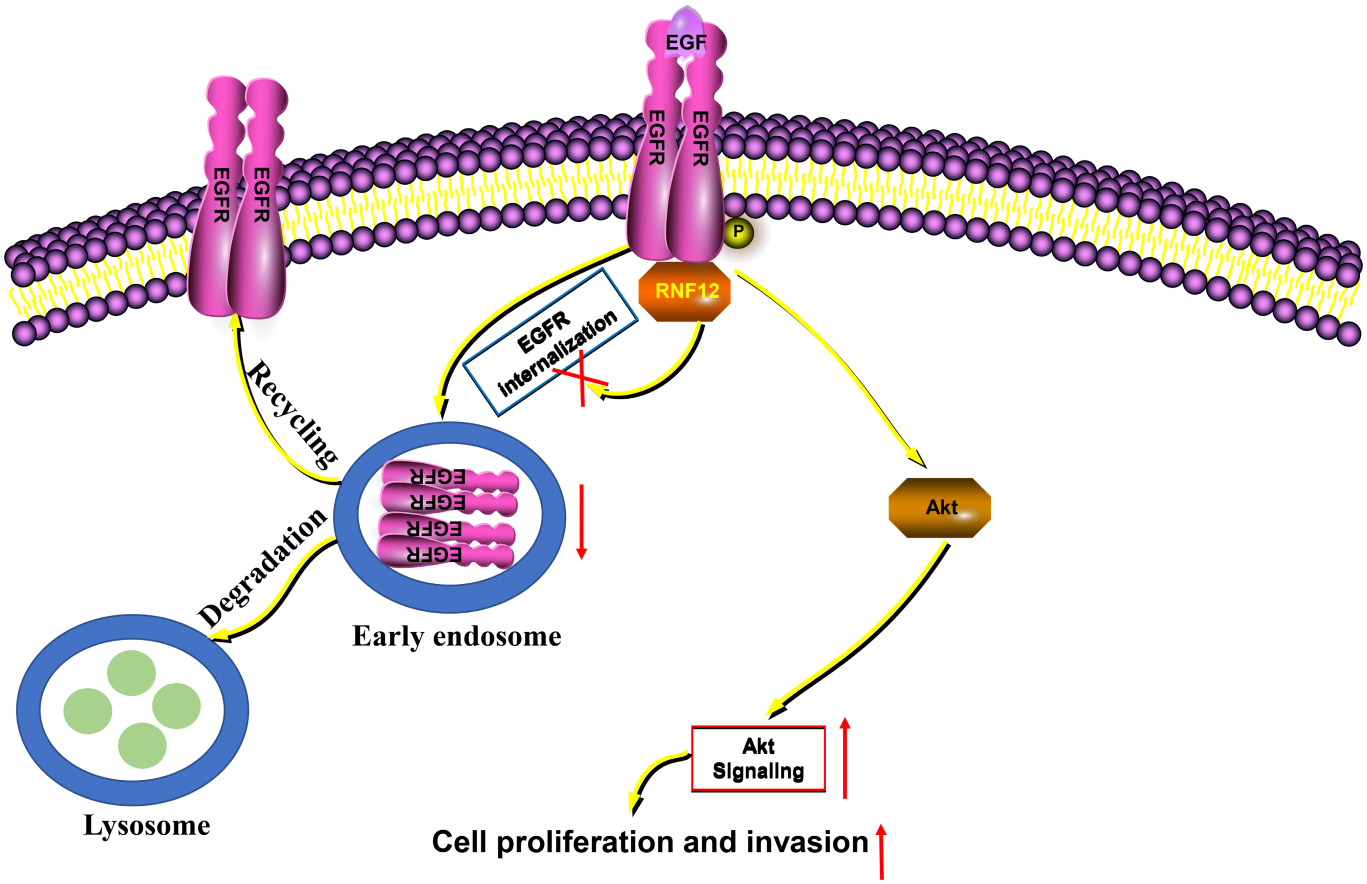
Plausible molecular mechanism. RNF12 could interact with EGFR and attenuate its internalization, which activates AKT signalling to promote cell proliferation and invasion.

In conclusion, our study shows that high RNF12 expression was correlated with worse clinicopathological features and a poor prognosis in liver cancer. RNF12 was found to activate PI3K‐AKT signalling to promote the progression of liver cancer by interacting with EGFR (Figure 8).

## AUTHOR CONTRIBUTIONS

**Chengpeng Yu:** Data curation (equal); methodology (equal); software (equal); writing – original draft (equal). **Dean Rao:** Data curation (equal); visualization (equal). **Tiantian Wang:** Formal analysis (equal); software (equal). **Jiaqi Sheng:** Visualization (equal). **Enjun Lv:** Data curation (equal). **Long Zhang:** Formal analysis (equal). **Xun Lu:** Formal analysis (equal). **Jingjing Yu:** Data curation (equal). **Huifang Liang:** Resources (equal); writing – review and editing (equal). **Jia Song:** Conceptualization (equal); investigation (equal). **Wenjie Huang:** Conceptualization (equal); project administration (equal); writing – review and editing (equal).

## FUNDING INFORMATION

This study was supported by grants from the National Natural Science Foundation of China (nos. 81871911 and 82173313 to W.H; 82103608 to J.S).

## CONFLICT OF INTEREST STATEMENT

The authors declare that they have no known competing financial interests or personal relationships that could have appeared to influence the work reported in this paper.

## ETHICS STATEMENT

Animal experiment (TJH‐202105003) and the use of clinical specimens (TJ‐IRB20220652) were reviewed and approved by the Research Ethics Committee of Tongji hospital of Huazhong University of Science and Technology (China).

## CONSENT

The patients/participants provided their written informed consent to participate in this study.

## CONSENT FOR PUBLICATION

Not applicable.

## Supporting information


FigureS1

FigureS2

FigureS3

FigureS4

TableS1

TableS2

TableS3

TableS4

## Data Availability

All supporting data are available in this study.
