# Supplementary figures and images for: Ring finger protein 12 activates AKT signalling to promote the progression of liver cancer by interacting with EGFR
Source: J Cell Mol Med. 2023 May 2;27(11):1523–38. doi: 10.1111/jcmm.17757 (PMC10243154; doi:10.1111/jcmm.17757)

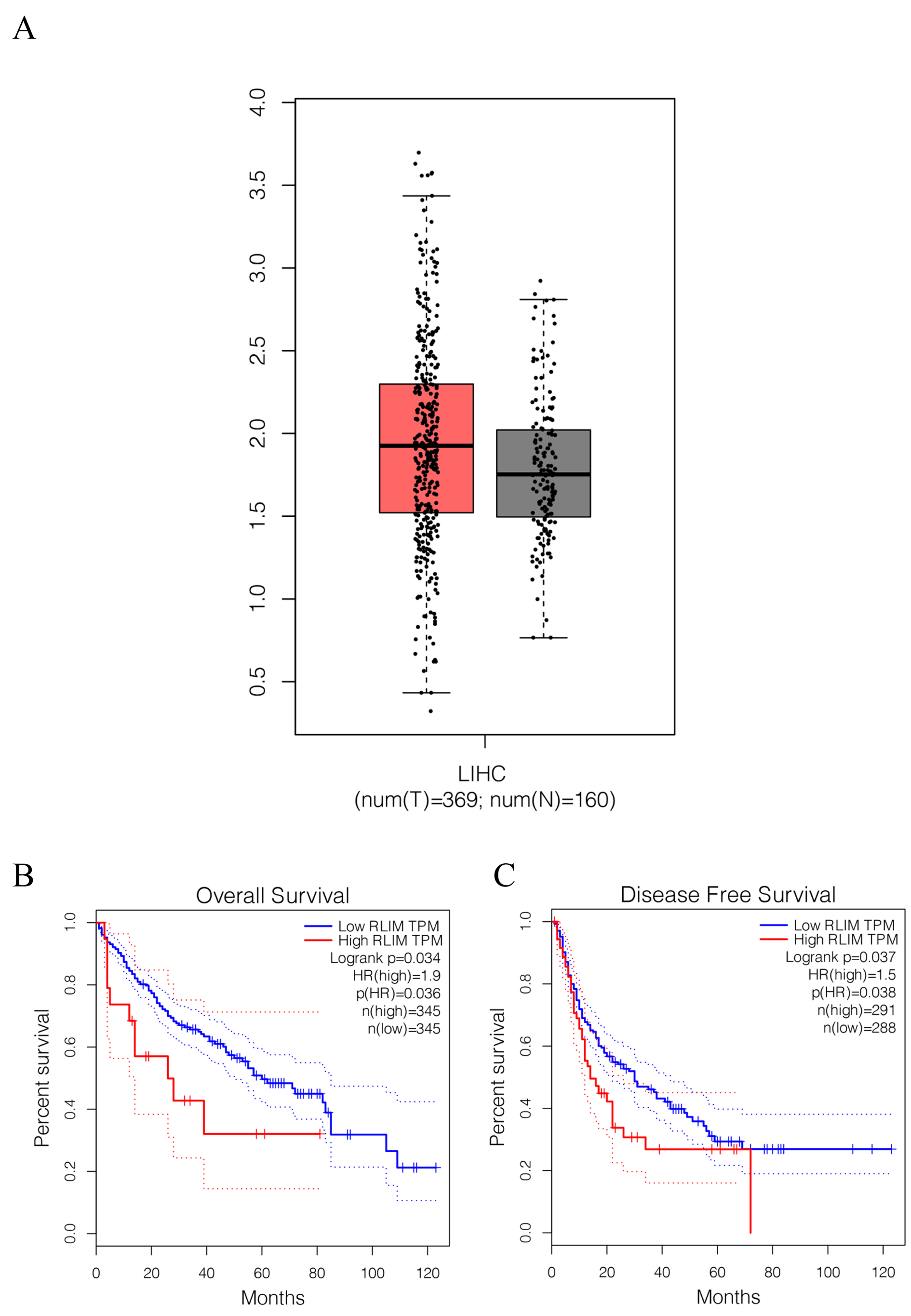

Supplement: Supplementary file 1 — FigureS1 [file JCMM-27-1523-s002.tif]

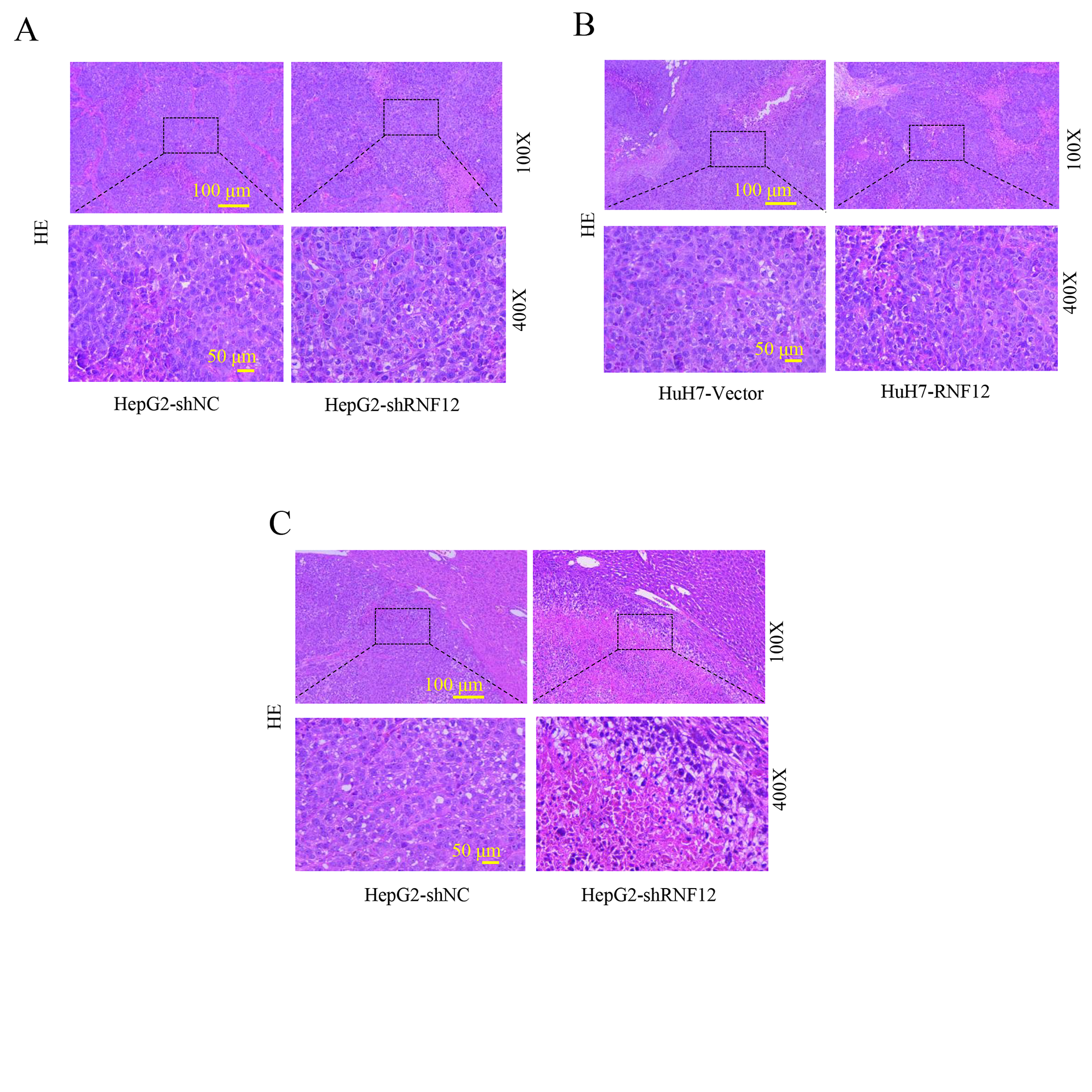

Supplement: Supplementary file 2 — FigureS2 [file JCMM-27-1523-s004.tif]

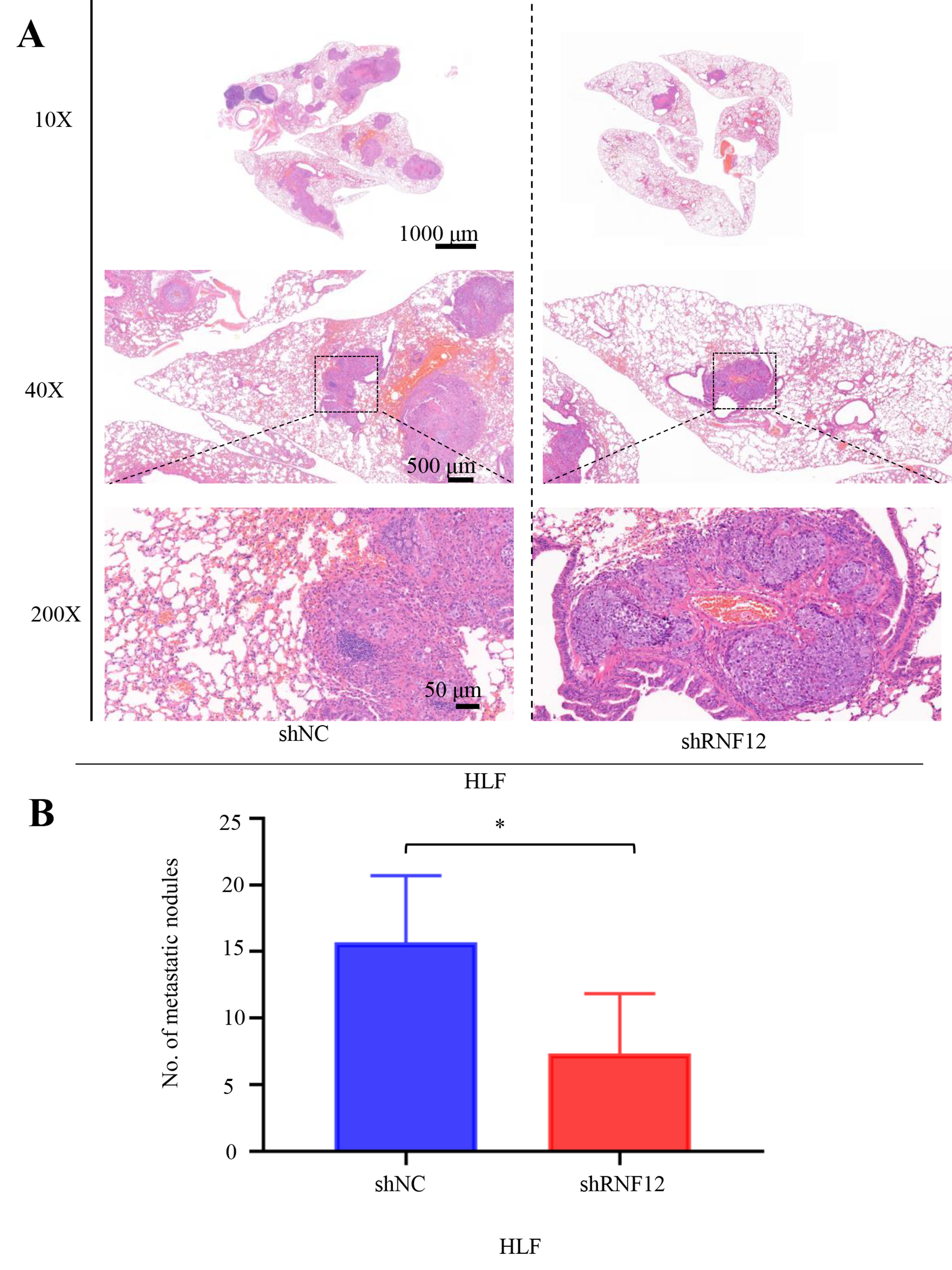

Supplement: Supplementary file 3 — FigureS3 [file JCMM-27-1523-s006.tif]

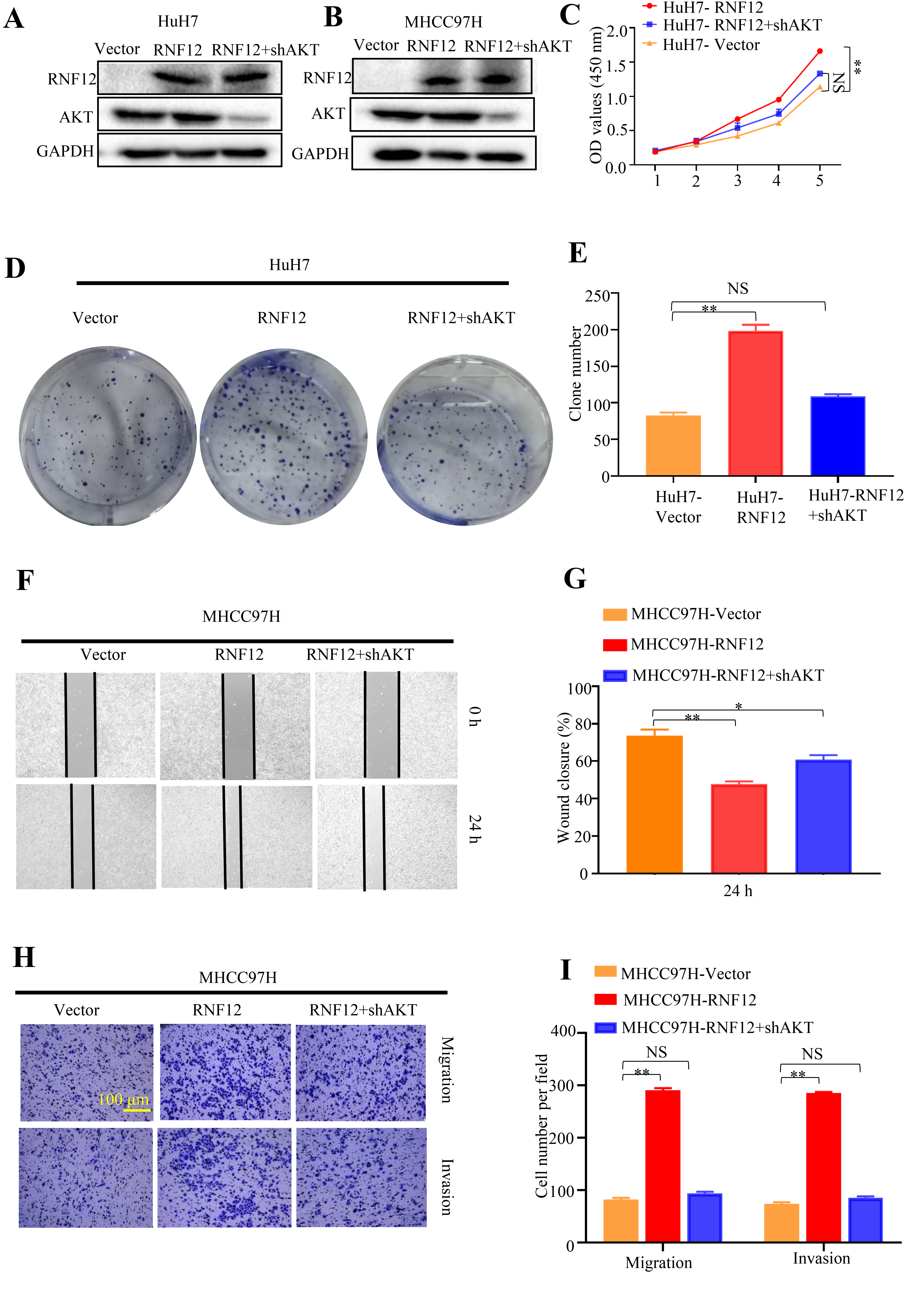

Supplement: Supplementary file 4 — FigureS4 [file JCMM-27-1523-s007.tif]
